# Calprotectin and Calgranulin C as Biomarkers of Pancreatic Tumors: Baseline Levels and Level Changes after Surgery

**DOI:** 10.1155/2019/6985703

**Published:** 2019-09-09

**Authors:** Michal Holub, Eva Bartáková, Alžběta Stráníková, Eva Koblihová, Simona Arientová, Marie Blahutová, Jan Máca, Miroslav Ryska

**Affiliations:** ^1^Department of Infectious Diseases, First Faculty of Medicine, Charles University and Military University Hospital Prague, 169 02 Praha 6, Czech Republic; ^2^Department of Clinical Microbiology, Military University Hospital Prague, 169 02 Praha 6, Czech Republic; ^3^Department of Surgery, Second Faculty of Medicine, Charles University and Military University Hospital Prague, 169 02 Praha 6, Czech Republic; ^4^Department of Clinical Biochemistry, Military University Hospital Prague, 169 02 Praha 6, Czech Republic; ^5^Department of Anesthesiology and Intensive Care Medicine, University Hospital of Ostrava, 17. Listopadu 1790/5, 708 52 Ostrava-Poruba, Czech Republic

## Abstract

Pancreatic tumors and their surgical resection are associated with significant morbidity and mortality, and the biomarkers currently used for these conditions have limited sensitivity and specificity. Because calprotectin and calgranulin C serum levels have been demonstrated to be potential biomarkers of certain cancers and complications of major surgery, the levels of both proteins were tested in the current study in patients with benign and malignant pancreatic tumors that were surgically removed. The baseline serum levels and kinetics of calprotectin and calgranulin C during the 7-day postoperative period were evaluated with immunoassays in 98 adult patients who underwent pancreatic surgery. The baseline serum levels of calprotectin and calgranulin C in patients with malignant (*n* = 84) and benign tumors (*n* = 14) were significantly higher (*p* < 0.01) when compared to those in the healthy controls (*n* = 26). The serum levels of both proteins were also significantly (*p* < 0.05) higher in patients with benign tumors than in those with malignant tumors. After surgery, the serum levels of calprotectin and calgranulin C were significantly (*p* < 0.01) higher than their baseline values, and this elevation persisted throughout the seven days of the follow-up period. Interestingly, starting on day 1 of the postoperative period, the serum levels of both proteins were significantly (*p* < 0.05) higher in the 37 patients who developed postoperative pancreatic fistulas (POPFs) than in the patients who had uneventful recoveries (*n* = 61). Moreover, the serum levels of calprotectin and calgranulin C demonstrated a significant predictive value for the development of POPF; the predictive values of these two proteins were better than those of the serum level of C-reactive protein and the white blood cell count. Taken together, the results of this study suggest that calprotectin and calgranulin C serum levels are potential biomarkers for pancreatic tumors, surgical injury to the pancreatic tissue and the development of POPFs.

## 1. Introduction

Calprotectin, also known as S100A8/9, and calgranulin C, also known as S100A12, are calcium-binding proteins stored in epithelial cells and phagocytes. Interestingly, in neutrophils, they account for 40% of the soluble cytosolic content. The intracellular roles of calprotectin and calgranulin C are associated with calcium regulation and cell motility. When these proteins are released extracellularly, they perform many actions, including stimulating neutrophil adhesion, migration, and release from the bone marrow and cytokine production by monocytes and epithelial cells. Calprotectin and calgranulin C have antibacterial and antiparasitic activities and have been shown to play roles in skin wound healing and liver and musculoskeletal regeneration [1]. Both proteins also stimulate proinflammatory activities and reactions to sterile injuries, as has been demonstrated after severe trauma and major surgery [2, 3]. Calprotectin and calgranulin C have been implicated in many human diseases, such as sepsis, Still's disease, rheumatoid arthritis, and laryngeal and pancreatic cancer [4–9].

Pancreatic cancer remains a highly lethal disease. The aggressive nature of the tumor and asymptomatic disease progression lead to delayed diagnosis and a high mortality rate. Pancreatic cancer has an estimated 20% resectability at the time of diagnosis, and the overall five-year survival rate is only 5% [10]. Surgery remains the only curative treatment. However, surgical treatment of pancreatic cancer is associated with significant morbidity and mortality during the early postoperative period, affecting 40–60% of patients [11]. Greenblatt et al. [12] reported that 27.1% of patients who underwent pancreatoduodenectomy suffered from a serious complication and 2.6% died within 30 days after the surgery. Hospital readmission after pancreatic resection is also common and can be attributed mostly to infectious complications. Currently used biomarkers that can predict postoperative complications, such as the white blood cell (WBC) count, C-reactive protein (CRP) serum levels, and amylase (AMS) levels in the pancreatic drainage fluid, have limitations. Recently, the associations of elevated calprotectin and calgranulin C serum levels after major elective gastrointestinal surgery with intensive care unit length of stay and 28-day and in-hospital mortality were demonstrated [13].

Thus, the aims of our study were to evaluate the serum levels of calprotectin and calgranulin C in adult patients undergoing surgery for malignant and benign pancreatic tumors and to test whether these proteins are better predictors of complications of pancreatic surgery than the biomarkers currently in use, including the CRP level, WBC count, and AMS in the pancreatic drainage fluid.

## 2. Methods

### 2.1. Patients

This prospective observational single-center study was performed in the Department of Surgery, Second Faculty of Medicine, Charles University and Military University Hospital Prague, Czech Republic. The study was approved by the Ethics Committee of the Military University Hospital Prague, and all study subjects signed informed consent forms before being enrolled in the study (reference number: 108/8-31/2015 ÚVN). Data were collected in the period between April 2015 and March 2017. All 113 enrolled patients were adults for whom pancreatic surgery was recommended by a multidisciplinary panel because of the suspicion of a malignant pancreatic tumor. Blood specimens were obtained the day before the elective surgery and then on days 1, 3, 5, and 7 after the surgery. The demographic and clinical characteristics are presented in Table 1. The postoperative complications were evaluated according to the 2016 update of the postoperative pancreatic fistula (POPF) guidelines, with two discrete grades of POPFs, namely, B and C. There were also two other cohorts: patients with uneventful recovery after the surgery and patients classified in the study as having grade A POPFs, which, according to the latest guidelines, are characterized as biochemical leaks or fistulas with no clinical impact [14]. The resected specimens were examined histopathologically according to the guidelines from the British Royal College of Pathologists regarding the definition of R1 (margin < 1 mm) following the Whipple procedure [15, 16] and standard lymphadenectomy [17]. For patients with tumors in the body or tail of the pancreas, left pancreatectomy and splenectomy were performed to ensure R0 resection [18]. We attempted to remove >15 lymph nodes to allow adequate pathological staging of the disease [19]. The control group consisted of 26 healthy volunteers (15 males and 11 females with a mean age of 51 years) who were recruited from the general population.

### 2.2. Laboratory Analysis

Blood samples for the WBC counts were collected into VACUETTE® K3 EDTA tubes (Greiner Bio-One GmbH, Frickenhausen, Germany) and processed immediately after collection. Blood samples for the analysis of the levels of CRP, calprotectin, and calgranulin C were collected into VACUETTE® Z Serum Clot Activator tubes (Greiner Bio-One GmbH). Serum CRP levels were measured immediately after sample collection, and the specimens used for the analysis of the calprotectin and calgranulin C levels were stored at -80°C. The serum levels of both proteins were measured with sandwich enzyme immunoassays (ELISAs) with the Evolis™ system (Stratec Biomedical Systems AG, Birkenfeld, Germany) and software from Bio-Rad Laboratories (Marnes-la-Coquette, France); the A100A8/A9 human ELISA and S100A12 human ELISA kits were provided by BioVendor (Brno, Czech Republic). The serum levels of CRP and the levels of AMS in the pancreatic drainage fluid were analyzed with the modular analyzer Cobas® 8000 (Roche Diagnostics GmbH, Manheim, Germany) using the CRPL3 and AMY-P kits (Roche Diagnostics GmbH). WBC counts were performed with the automated hematology system Sysmex XE-5000 (Sysmex Europe GmbH, Norderstedt, Germany).

### 2.3. Statistical Analysis

The analysis of differences in the baseline values among groups of patients and the healthy controls was performed with a Kruskal-Wallis ANOVA accompanied by a post hoc Wilcoxon test or a set of post hoc Wilcoxon tests with *p* values adjusted for multiple comparisons by Holm's method. To compare groups of patients to the normative values, one-sided Wilcoxon tests were used, with the *p* values adjusted for multiple comparisons by Holm's method. Differences in gender among groups of patients and healthy controls were studied using a *χ*^2^ test. The analysis of differences among the groups of patients with grade A, B, and C complications was also performed with a Kruskal-Wallis ANOVA accompanied by a set of post hoc Wilcoxon tests with *p* values adjusted for multiple comparisons by Holm's method. To compare groups of patients with complications and healthy controls or patients without complications, a Wilcoxon test was used. The *p* values were also adjusted for multiple comparisons by Holm's method. A logistic regression model was used to evaluate the risk of complications with respect to other parameters. For the correlation analyses, Spearman's correlation coefficient was used. All analyses were performed by the certified biostatistician Dr. Václav Čapek with R statistical software, version 3.4.2., R Core Team (Vienna, Austria). *p* values less than 5% were considered statistically significant.

## 3. Results

### 3.1. Baseline Serum Levels of Calprotectin and Calgranulin C

The baseline serum levels of calprotectin and calgranulin C were evaluated in an initial cohort of 98 patients who either had benign pancreatic tumors (i.e., serous cystadenomas or pancreatic pseudocysts) or histologically confirmed malignant pancreatic tumors, including ductal adenocarcinoma, neuroendocrine tumors, and mucinous cystadenocarcinoma; 15 patients with biliary carcinoma, inconclusive histology findings, intraductal papillary mucinous neoplasia, or another type of tumor were excluded from the evaluation. The levels of calprotectin and calgranulin C were significantly higher in patients with benign and malignant pancreatic tumors than in the healthy controls, and the calprotectin serum levels were also significantly higher in patients with benign tumors than in patients with malignant tumors. In addition, the patients with malignant tumors were significantly older than the healthy controls and the patients with benign tumors, and no differences were observed between the cohorts with malignant and benign tumors with regard to their baseline serum CRP levels, WBC counts, and reference values. These data are shown in Table 2.

### 3.2. Kinetics of Calprotectin, Calgranulin C, and Routine Biomarkers after Surgery

**Figure 1:**
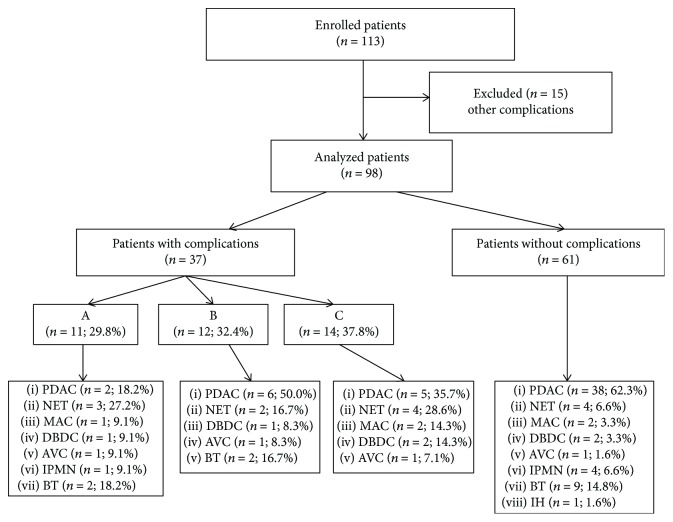
Distributions of malignant and benign tumors among patients with evaluated biomarker kinetics (PDAC: pancreatic ductal adenocarcinoma; NET: pancreatic neuroendocrine tumor; MAC: mucinous cystadenocarcinoma; AVC: ampulla of Vater carcinoma; DBDC: distal bile duct carcinoma; IPMN: intraductal papillary mucinous neoplasm; BT: benign tumor; IH: inconclusive histology).

The serum levels of calprotectin, calgranulin C, and routinely used biomarkers were evaluated in the postoperative period in an initial cohort of 98 patients either with an uncomplicated postoperative course or with grade A, B, or C POPFs; 15 patients with complications other than POPFs were excluded from this evaluation. The evaluated patients did not significantly differ in terms of age, and the distributions of malignant and benign tumors were proportional among the groups (Figure 1). The serum levels of calprotectin and calgranulin C were significantly higher in patients with surgical complications than in patients with uneventful recoveries from day 1 to day 7 of the postoperative period. Furthermore, on day 1 of the postoperative period, the serum levels of calgranulin C were significantly higher in patients who developed grade B POPFs than in patients who developed grade A (*p* = 0.008) and C (*p* = 0.029) POPFs, and the serum levels of calprotectin were significantly higher in patients with grade B POPFs than in patients with grade A POPFs (*p* = 0.039). The serum levels of calprotectin and calgranulin C during the study period are shown in Table 3.

The routine biomarkers analyzed included the WBC count, CRP level, and AMS level in the pancreatic drainage fluid. When compared to the WBC count in patients with an uncomplicated postoperative course, the WBC count was significantly higher in patients with grade B POPFs on days 3, 5, and 7 and in patients with grade A POPFs on day 7. Furthermore, on days 3, 5, and 7, the serum CRP levels were significantly higher in patients with grade B and C POPFs than in the patients with uneventful recoveries. AMS levels in the pancreatic drainage fluid were significantly higher from day 1 to day 7 in all three groups with postoperative complications than in the group of patients with uncomplicated postoperative courses. These results are shown in Table 3. Furthermore, on day 3, the WBC count was significantly higher in patients with grade B POPFs than in patients with grade C POPFs (*p* = 0.019), and the serum CRP levels were significantly higher in patients with grade B (*p* = 0.017) and grade C (*p* = 0.026) POPFs than in patients with grade A POPFs. The serum CRP levels on day 7 were significantly higher in patients with grade C POPFs than in patients with grade A POPFs (*p* = 0.038). Apart from these differences, the WBC count, serum CRP levels, and AMS levels in the pancreatic drainage fluid did not differ significantly among the groups of patients with grade A, B, and C POPFs.

### 3.3. Logistic Regression Analysis

The serum levels of calprotectin and calgranulin C demonstrated predictive values for surgical complications that were better than those of the CRP level and WBC count but worse than that of the level of AMS in the pancreatic drainage fluid. The results of the logistic regression analysis and the area under the curve (AUC), cutoff values, sensitivities, and specificities are shown in Table 4.

### 3.4. Correlations of Calprotectin and Calgranulin C Levels with the WBC Counts, Serum CRP Levels, and AMS Levels in the Pancreatic Drainage Fluid on Days 1, 3, 5, and 7

The calprotectin serum levels were significantly correlated with the WBC count, CRP levels, and AMS levels on days 3 and 5 and with the WBC count and the CRP levels on day 7. The calgranulin C serum levels were significantly correlated with only AMS on days 3 and 5. The results are shown in Table 5.

## 4. Discussion

In this study, we found elevated baseline serum levels of calprotectin and calgranulin C in patients with malignant and benign pancreatic tumors and the upregulation of those levels after surgery and in patients with postoperative complications.

**Figure 2:**
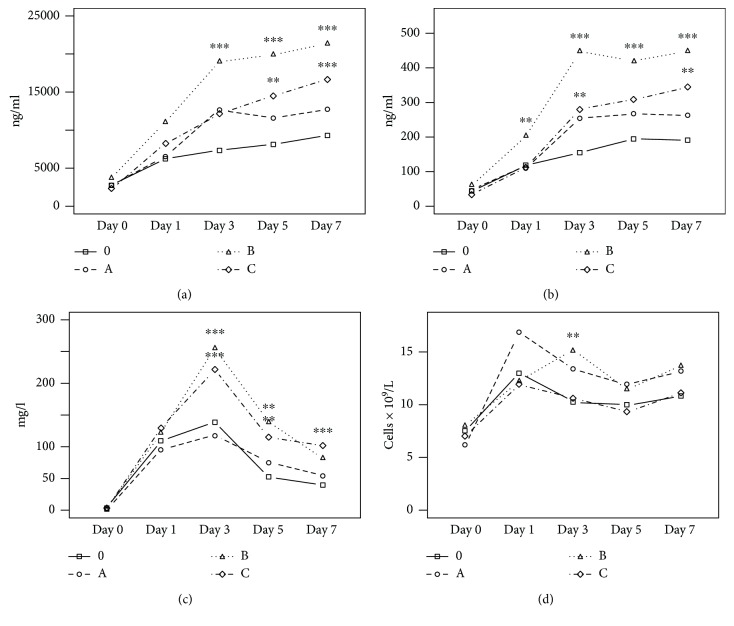
Kinetics of calprotectin (a), calgranulin C (b), C-reactive protein (c), and white blood cells (d) during the study period (0: uncomplicated course; A: POPF grade A; B: POPF grade B; C: POPF grade C; ^∗∗^*p* < 0.01; ^∗∗∗^*p* < 0.001).

We observed increased serum levels of calprotectin and calgranulin C after pancreatic surgery, which persisted throughout the seven days of the study period. Similarly, Máca et al. [3] found persistent elevations of the serum levels of S100A8 (the homodimer of the calprotectin heterodimer complex) and calgranulin C for three days after gastrointestinal tract surgery; these levels were significantly higher than those observed in healthy volunteers. Unlike in the study by Máca et al., which did not report the baseline values of the investigated alarmins, we were able to evaluate the direct effect of surgical injury on the serum levels of calprotectin and calgranulin C because we measured the baseline concentrations of both proteins. Two- to fourfold increases in the serum levels of calprotectin and calgranulin C were observed 24 hours after surgery, indicating the potential of both proteins to serve as markers of the intensity of the sterile surgical injury. Interestingly, the kinetics of the calprotectin and calgranulin C serum levels seemed to be influenced by postoperative complications, with the highest concentrations observed in patients who developed grade B POPFs. This finding may reflect the irritation of the pancreatic tissue by the pancreatic fluid and/or persistent inflammation, which are associated with the development of POPFs. In addition, the prognostic value of the calprotectin and calgranulin C serum levels seems to be limited by the observed trend of higher concentrations in patients who developed grade B POPFs than in those who developed grade C POPFs, which are associated with the most severe complications such as reoperation, organ failure, and death [14]. However, a similar finding was reported in patients who had suffered severe blunt trauma complicated with secondary bacterial infections; those patients had calprotectin serum levels that were lower than those in patients with uneventful recoveries [2]. Although the AMS levels in the pancreatic drainage fluid are very sensitive for the prediction of the development of POPFs, the serum levels of calprotectin and calgranulin C may be additional useful parameters, with better prognostic values than the currently used routine blood biomarkers, such as the WBC count and CRP serum levels (Figure 2). However, the importance of the blood biomarkers is particularly marked in patients at the time of the removal of the pancreatic drain, which is usually removed 5-7 days after the surgery. Interestingly, at that time, the serum levels of calprotectin and calgranulin C demonstrated the best diagnostic sensitivity for the development of POPFs in our study. Therefore, this observation may suggest that the close monitoring of the serum levels of calprotectin and calgranulin C after the removal of the pancreatic drain may aid in the detection of the development of POPFs. To the best of our knowledge, this is an original observation that has not yet been reported.

**Figure 3:**
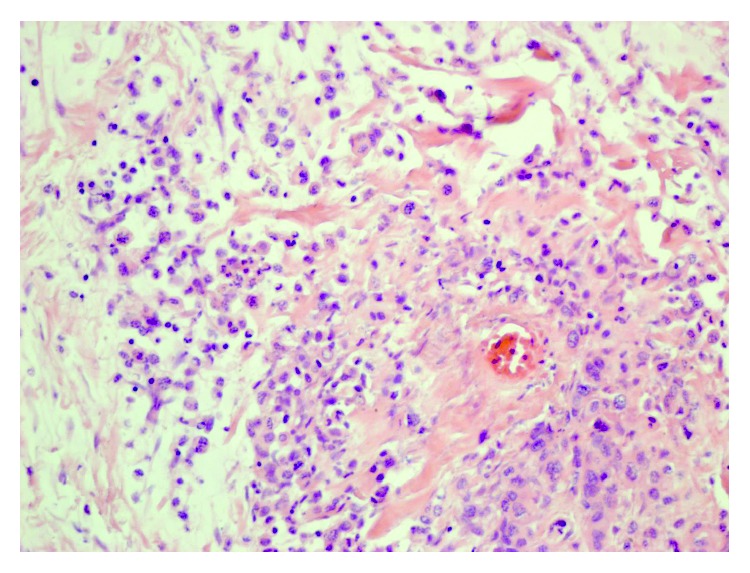
Histological image of ductal adenocarcinoma (hematoxylin-eosin staining, magnification 200x).

**Figure 4:**
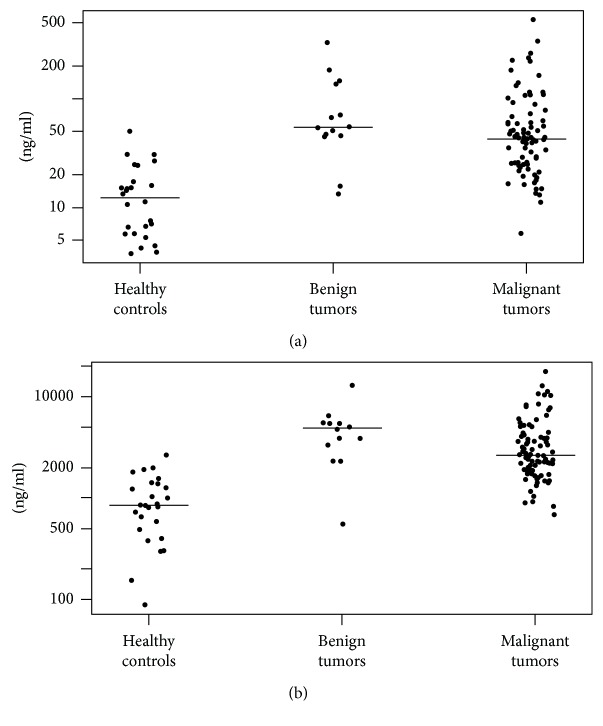
Calprotectin (a) and calgranulin C (b) baseline levels in patients with pancreatic tumors and healthy controls.

It is well known that S100A proteins play roles in pancreatic cancer progression and metastasis [20]. However, most attention has been paid to proteins S100A2, S100A4, S100A6, and S100A11, which are involved in the degradation of the extracellular matrix and metastasis, and only a few studies have investigated the roles of calprotectin and calgranulin C. In patients with pancreatic ductal adenocarcinoma with diabetes mellitus, Moz et al. [21] demonstrated that an increased tissue expression level of S100A8 is associated with chronic pancreatitis and not with ductal adenocarcinoma, whereas a decreased expression level of S100A9 is correlated with a poor outcome for patients with ductal adenocarcinoma. Similarly, we observed the highest calprotectin serum levels in patients with benign pancreatic tumors, indicating that inflammation is a major inducer of the release of calprotectin into the systemic circulation. It is worth noting that the observed difference between the patients with benign and malignant pancreatic tumors cannot be explained by the higher median age of the patients with cancer because the reference ranges for the serum calprotectin levels increase with increasing age [22, 23]. Furthermore, compared with the healthy controls, the patients with malignant pancreatic tumors enrolled in our study also had significantly higher baseline serum calprotectin and calgranulin C levels. This finding probably reflects the local secretion of both proteins from the malignant tissue. Since malignant pancreatic tumors are frequently associated with lymphoplasmocytic infiltrate and neutrophils are scarce in the tissue (Figure 3), the source of both proteins is probably the cancer cells. This notion can be supported by finding of the overexpression of S100A8 and S100A9 by the cancer cells in renal cell carcinoma [24]. Importantly, elevated levels of S100A8 and S100A9 were found in the ductal fluid of patients with pancreatic cancer, and the high expression levels of both proteins were associated with a median survival time that was nearly halved in comparison with that of patients without similarly elevated levels. This finding in the context of our observation may suggest a detrimental effect of pancreatic tumor-induced upregulation of calprotectin and calgranulin C [25]. Another explanation for the elevated serum calprotectin and calgranulin C levels could be biliary obstruction, which is a common finding in patients with pancreatic tumors. This notion can be supported by the observation of the high biliary levels of S100A8/9 in patients with primary sclerosing cholangitis [26]. The observed elevations are interesting, especially in the context of the normal values of the routinely used biomarkers in enrolled patients. Although calprotectin and calgranulin C serum levels do not seem to be highly specific for individual pancreatic pathologies (Figure 4), their release into the blood, which is not followed by the stimulation of systemic inflammation, indicates the potential for the use of both proteins as markers of local inflammation or progression to malignancy. Moreover, the persistent elevation of the levels of calprotectin and calgranulin C in the blood may suggest a risk of progression from benign to malignant pancreatic tumors. This suggestion is supported by the finding that upregulation of the S100A8 or S100A9 proteins may promote tumorigenesis [27].

**Figure 5:**
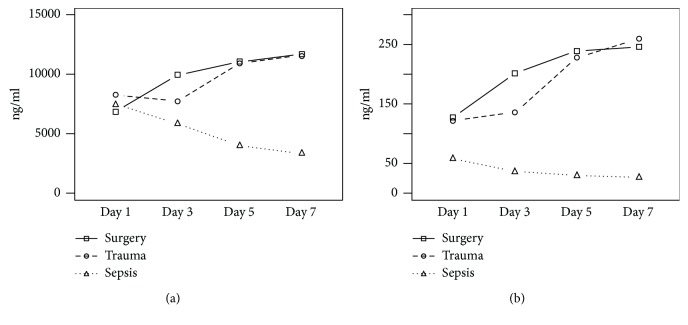
Comparison of calprotectin and calgranulin C kinetics after surgery, trauma, and the initiation of antibiotic therapy for sepsis, which was continued through day 7.

Both calprotectin and calgranulin C are well-known indicators of systemic inflammation, and their levels have been found to be elevated in the blood in patients with a diverse range of inflammatory and infectious conditions, including acute otitis media, familial Mediterranean fever, inflammatory bowel disease, Kawasaki's disease, cystic fibrosis, rheumatoid and psoriatic arthritis, tuberculosis, severe sepsis, ventilator-associated lung injury, trauma, noninfectious critical illness, and microtrauma induced by long-distance running [28–33]. Furthermore, surgery and trauma are also associated with elevated serum levels of calprotectin and calgranulin C. In trauma patients, the high serum levels of calprotectin and calgranulin C are positively correlated with a severe course as indicated by the Sequential Organ Failure Assessment score and elevated CRP levels in the early posttraumatic period [32]. Similarly, in patients undergoing major abdominal surgery, the levels of calgranulin C and the S100A8 protein were elevated three days after the surgery, and the serum levels of those proteins were positively correlated with the length of stay in the intensive care unit, in-hospital and 28-day mortality, and increased CRP serum levels [3, 13]. In our study, the highest CRP serum levels and WBC counts were also observed in the early postoperative period. As could have been expected, the high serum CRP levels at day 3 were associated with the development of grade B and C POPFs [34]. The WBC count demonstrated similar kinetics in the patients with grade A and B POPFs, whereas the patients with grade C POPFs had WBC counts similar to those of the patients with uneventful recoveries. While elevated serum levels of CRP probably reflect the activation of systemic inflammation elicited by the surgical injury, leukocytosis in the patients with grade B POPFs is usually associated with a mild infection [14]. Furthermore, a trend towards persistent leukocytosis in the patients with grade A POPFs together with a relatively low WBC count in the patients with grade C POPFs suggests that the WBC count is not a reliable predictor of the development of POPFs, which has been already demonstrated [34]. In addition, the persistent elevation of the serum levels of calprotectin and calgranulin C together with the decrease in serum CRP levels observed in the patients with uneventful recoveries and grade A and B POPFs after day 3 may indicate the presence of local inflammation without the stimulation of the systemic response or may reflect the action of calprotectin and calgranulin C in wound healing and pancreatic tissue regeneration [1]. This interpretation can be supported by the fact that the kinetics of both proteins after severe trauma and pancreatic surgery are the inverse of the kinetics observed in bacterial sepsis after the suppression of infection by antimicrobial therapy (Figure 5) [23]. On the other hand, the highest CRP serum levels observed on day 7 in the patients with grade C POPFs were not fully associated with a similarly intense increase in the serum levels of calprotectin and calgranulin C, which may suggest that the neutrophils—the major source of both proteins—are already exhausted and their ability to mount a response to a secondary injury is compromised.

The study had several limitations. First, the study was performed in a single center, and its monocentric nature together with the laboratory immunoassays utilized limits the direct translation of the findings to routine use. However, a new analytical method for measuring serum levels of calprotectin has recently been introduced into routine practice, which means that additional data could easily be gathered to verify our findings. Second, the primary aim of the study was to evaluate the utility of the calprotectin and calgranulin C serum levels as biomarkers of surgical injury, and the analysis of the baseline levels of the two proteins in benign and malignant pancreatic tumors was performed in a post hoc manner. Third, the groups of patients with certain grades of postoperative complications were relatively small and were not homogenous regarding the type and extent of pancreatic surgery; therefore, the findings must be interpreted with caution. Lastly, we could not assess the effects on mortality, because all evaluated patients were discharged from the hospital in stable conditions, and the only deaths in the postoperative period occurred among the patients with complications other than POPFs.

In conclusion, it is clear that calprotectin and calgranulin C are upregulated after pancreatic surgery and, to a lesser extent, in benign and malignant pancreatic tumors. This observation, together with the finding of the highest concentrations of both investigated alarmins in patients with postoperative complications, may suggest the potential usefulness of calprotectin and calgranulin C as biomarkers. Because pancreatic cancer and surgery are severe conditions and the current biomarkers have inherent limitations, further studies on calprotectin and calgranulin C should be performed.

## Figures and Tables

**Table 1:** Demographic and clinical data of the study group.

Patients	*n* = 113			
Age in years (mean)	61.4			
Gender (male/female)	56/57			

Conditions	*n*	%	Age	M/F
Ductal adenocarcinoma	60	53.1	64.4	26/34
Neuroendocrine tumor	16	14.2	58	9/7
Benign tumor	14	12.4	50.9	7/7
Intraductal papillary mucinous neoplasm	6	5.3	63.8	4/2
Mucinous cystadenocarcinoma	5	4.4	55.8	1/4
Distal bile duct carcinoma	6	5.3	66.6	5/1
Ampulla of Vater carcinoma	4	3.5	62.5	2/2
Inconclusive histology	1	0.9	62.1	1/0
Another tumor type	1	0.9	64	1/0

Operations				
Pancreatoduodenectomy	64	56.6	61.9	32/32
Distal pancreatectomy	28	24.8	62.1	12/16
Palliative procedures	8	7.1	61	5/3
Total pancreatectomy	9	8.0	57.6	5/4
Other	4	3.5	58	2/2

Complications				
None	61	54.0	59.4	29/32
Complication grade A	11	9.7	65.3	7/4
Complication grade B	12	10.6	65.2	5/7
Complication grade C	14	12.4	62.1	10/4
Other	15	13.3	63	5/10

**Table 2:** Baseline characteristics of patients with malignant and benign tumors and healthy controls and their comparisons.

	Healthy controls (*n* = 26)	Patients with malignant tumors (*n* = 84)	Patients with benign tumors (*n* = 14)	*p*
Gender (M/F)	15/11	37/47	7/7	
Mean age (years)	50.9	62.9^∗∗^	50.9	<0.05
Calprotectin (ng/mL)	847 (510-1349)	2650 (1885-4233)^∗∗^	4914 (3486-5478)^∗∗^	<0.05
Calgranulin C (ng/mL)	12.3 (6.0-16.9)	42.6 (25.0-64.3)^∗∗^	54.7 (46.2-120.3)^∗∗^	N.s.

*p*: difference between cohorts with benign and malignant tumors; M: male; F: female; N.s.: not significant. ^∗^*p* < 0.05 and ^∗∗^*p* < 0.01.

**Table 3:** The comparisons of the level of AMS in the pancreatic drainage fluid; the levels of calprotectin, calgranulin C, and CRP in the serum; and the WBC count in patients with POPF grades A, B, and C and those with uncomplicated postoperative courses.

	0	A	B	C
*Calprotectin (ng/mL)*				
Day 0	2731 (1705-4781)	2681 (2289-3017)	3714 (2284-4550)	2274 (1597-3558)
Day 1	6233 (4354-8586)	6540 (5617-7630)	11065 (6808-12425)^∗^	8253 (5336-10856)
Day 3	7337 (4940-11306)	12649 (7549-19181)^∗^	18983 (15364-22685)^∗∗^	12087 (8930-15833)^∗^
Day 5	8118 (5671-11794)	11569 (9442-20617)	19898 (13254-23208)^∗∗^	14496 (11787-15597)^∗∗^
Day 7	9309 (5148-12605)	12735 (10835-21866)^∗^	21358 (14284-24551)^∗∗^	16643 (12150-19353)^∗∗^

*Calgranulin C (ng/mL)*				
Day 0	42.5 (19.7-60.6)	50.8 (38.8-54.1)	60.4 (24.7-96.6)	34.3 (25.2-48.2)
Day 1	118.6 (76.8-165.1)	108.8 (97.0-146.2)	202.9 (155.5-242.7)^∗∗^	112.1 (97.9-155.7)
Day 3	154.6 (93.4-235.1)	254.7 (158.7-402.2)^∗^	447.3 (330.8-542.6)^∗∗^	279.8 (229.4-334.9)^∗∗^
Day 5	195.0 (114.5-259.7)	266.9 (144.2-453.8)	419.2 (294.9-647.5)^∗∗^	308.8 (214.3-379.3)^∗^
Day 7	191.1 (111.0-276.0)	262.6 (204.1-450.3)^∗^	448.8 (301.1-581.0)^∗∗^	343.8 (275.0-359.0)^∗∗^

*WBC (10^9^ cells/L)*				
Day 0	7.5 (5.9-9)	6.2 (5.4-7.7)	8.0 (6.8-9.1)	7.0 (6.6-7.9)
Day 1	13.0 (11.6-15.5)	16.8 (12.6-18.1)	12.2 (10.6-19.2)	11.9 (11.5-13.2)
Day 3	10.2 (8.0-12.9)	13.4 (12.4-14.5)	15.1 (12.8-21.4)^∗∗^	10.5 (8.3-12.5)
Day 5	10.0 (7.6-11.3)	11.8 (10.1-14.0)	11.5 (10.4-15.6)^∗^	9.3 (7.3-11.1)
Day 7	10.8 (8.6-12.5)	13.2 (11.2-16.7)^∗^	13.6 (11.7-16.9)^∗^	11.1 (9.3-12.9)

*CRP (mg/L)*				
Day 0	3.9 (2.0-11.5)	1.0 (0.9-6.3)	3.9 (1.8-5.0)	4.0 (2.0-6.5)
Day 1	109.6 (72.1-129.2)	95.0 (87.4-102.0)	122.2 (104.6-143.8)	129.7 (102.0-154.1)
Day 3	138.9 (88.7-171.5)	118.0 (70.9-213.1)	255.5 (190.1-287.7)^∗∗^	221.9 (160.8-249.6)^∗∗^
Day 5	52.5 (38.2-86.5)	75.3 (47.8-122.0)	138.6 (86.7-193.2)^∗∗^	114.8 (89.5-190.0)^∗∗^
Day 7	40.0 (26.6-71.0)	54.3 (31.4-77.6)	81.9 (52.4-126.1)^∗^	101.9 (79.7-144.0)^∗∗^

*AMS (μkat/L)*				
Day 0	N.d.	N.d.	N.d.	N.d.
Day 1	2.3 (0.7-18.8)	18.4 (7.1-53.1)^∗^	48.3 (19.9-169.0)^∗∗^	102.3 (39.8-186.5)^∗∗^
Day 3	0.8 (0.2-1.9)	9.3 (3.6-24.0)^∗∗^	12.0 (4.7-33.6)^∗∗^	3.1 (1.6-19.5)^∗∗^
Day 5	0.3 (0.1-0.6)	1.1 (0.8-2.7)^∗∗^	9.0 (2.6-26.9)^∗∗^	0.9 (0.3-61.4)^∗∗^
Day 7	0.3 (0.1-0.7)	5.2 (1.6-67.9)^∗^	14.8 (9.9-40.1)^∗^	13.5 (9.5-57.7)^∗^

WBC: white blood cell; CRP: C-reactive protein; AMS: amylase; 0: uncomplicated postoperative course; A, B, C: grades of postoperative pancreatic fistulas; N.d.: not determined; ^∗^*p* < 0.05 and ^∗∗^*p* < 0.01.

**Table 4:** AUCs, cutoff values, sensitivities, and specificities of the biomarkers for the prediction of surgical complications.

	AUC	Threshold	Sensitivity	Specificity
*Day 1*				
Calprotectin (ng/mL)	0.6324	6177.25	0.5000	0.7297
Calgranulin C (ng/mL)	0.6072	97.66	0.4333	0.8378
WBC count (cells × 10^9^/L)	0.5268	12.05	0.6833	0.4595
CRP (mg/L)	0.5747	129.65	0.7755	0.4118
AMS (*μ*kat/L)	0.8240	7.67	0.6842	0.8621

*Day 3*				
Calprotectin (ng/mL)	0.7780	9987.50	0.7049	0.8108
Calgranulin C (ng/mL)	0.7873	182.12	0.6230	0.8649
WBC count (cells × 10^9^/L)	0.6645	10.72	0.5763	0.7297
CRP (mg/L)	0.7151	206.30	0.8621	0.5676
AMS (*μ*kat/L)	0.8429	3.77	0.8974	0.6571

*Day 5*				
Calprotectin (ng/mL)	0.7707	11049.5	0.7000	0.8108
Calgranulin C (ng/mL)	0.7423	254.80	0.7500	0.7297
WBC count (cells × 10^9^/L)	0.6289	13.81	0.9322	0.2941
CRP (mg/L)	0.7638	76.30	0.6964	0.7879
AMS (*μ*kat/L)	0.8171	0.62	0.7632	0.7742

*Day 7*				
Calprotectin (ng/mL)	0.8163	11693.25	0.7037	0.7838
Calgranulin C (ng/mL)	0.7815	297.50	0.8148	0.6757
WBC count (cells × 10^9^/L)	0.6694	14.98	0.9348	0.3824
CRP (mg/L)	0.7216	51.70	0.6136	0.8125
AMS (*μ*kat/L)	0.8852	0.94	0.8182	0.9474

AUC: area under the ROC curve; WBC: white blood cell; CRP: C-reactive protein; AMS: amylase.

**Table 5:** Correlations of calprotectin and calgranulin C serum levels with the CRP levels, WBC counts, and AMS levels in the pancreatic drainage fluid.

	WBC	CRP	AMS
*Day 1*			
Calprotectin	0.0913	0.1422	0.1713
Calgranulin C	0.0918	0.0157	0.1385

*Day 3*			
Calprotectin	0.4375^∗^	0.6020^∗^	0.5202^∗^
Calgranulin C	0.4128	0.5126	0.5571^∗^

*Day 5*			
Calprotectin	0.4532^∗^	0.6659^∗^	0.4141^∗^
Calgranulin C	0.4192	0.5745	0.4059^∗^

*Day 7*			
Calprotectin	0.4950^∗^	0.6023^∗^	0.0652
Calgranulin C	0.4644	0.5466	-0.0372

WBC: white blood cell; CRP: C-reactive protein; AMS: amylase. ^∗^Correlation coefficient (*r*) with *p* < 0.01.

## Data Availability

The data used to support the findings of this study are included within the article.
